# Development, comparison, and validation of four intelligent, practical machine learning models for patients with prostate-specific antigen in the gray zone

**DOI:** 10.3389/fonc.2023.1157384

**Published:** 2023-06-08

**Authors:** Taobin Liu, Xiaoming Zhang, Ru Chen, Xinxi Deng, Bin Fu

**Affiliations:** ^1^ Department of Urology, The First Affiliated Hospital of Nanchang University, Nanchang, China; ^2^ Jiangxi Institute of Urology, Nanchang, Jiangxi, China; ^3^ Department of Urology, Jiu Jiang NO.1 People's Hospital, Jiujiang, China

**Keywords:** prostate cancer, gray area PSA, machine learning, LogisticRegression, XGBoost, GaussianNB, LGBMClassifier

## Abstract

**Purpose:**

Machine learning prediction models based on LogisticRegression, XGBoost, GaussianNB, and LGBMClassifier for patients in the prostate-specific antigen gray zone are to be developed and compared, identifying valuable predictors. Predictive models are to be integrated into actual clinical decisions.

**Methods:**

Patient information was collected from December 01, 2014 to December 01, 2022 from the Department of Urology, The First Affiliated Hospital of Nanchang University. Patients with a pathological diagnosis of prostate hyperplasia or prostate cancer (any PCa) and having a prostate-specific antigen (PSA) level of 4–10 ng/mL before prostate puncture were included in the initial information collection. Eventually, 756 patients were selected. Age, total prostate-specific antigen (tPSA), free prostate-specific antigen (fPSA), fPSA/tPSA, prostate volume (PV), prostate-specific antigen density (PSAD), (fPSA/tPSA)/PSAD, and the prostate MRI results of these patients were recorded. After univariate and multivariate logistic analyses, statistically significant predictors were screened to build and compare machine learning models based on LogisticRegression, XGBoost, GaussianNB, and LGBMClassifier to determine more valuable predictors.

**Results:**

Machine learning prediction models based on LogisticRegression, XGBoost, GaussianNB, and LGBMClassifier exhibit higher predictive power than individual metrics. The area under the curve (AUC) (95% CI), accuracy, sensitivity, specificity, positive predictive value, negative predictive value, and F1 score of the LogisticRegression machine learning prediction model were 0.932 (0.881–0.983), 0.792, 0.824, 0.919, 0.652, 0.920, and 0.728, respectively; of the XGBoost machine learning prediction model were 0.813 (0.723–0.904), 0.771, 0.800, 0.768, 0.737, 0.793 and 0.767, respectively; of the GaussianNB machine learning prediction model were 0.902 (0.843–0.962), 0.813, 0.875, 0.819, 0.600, 0.909, and 0.712, respectively; and of the LGBMClassifier machine learning prediction model were 0.886 (0.809–0.963), 0.833, 0.882, 0.806, 0.725, 0.911, and 0.796, respectively. The LogisticRegression machine learning prediction model has the highest AUC among all prediction models, and the difference between the AUC of the LogisticRegression prediction model and those of XGBoost, GaussianNB, and LGBMClassifier is statistically significant (p < 0.001).

**Conclusion:**

Machine learning prediction models based on LogisticRegression, XGBoost, GaussianNB, and LGBMClassifier algorithms exhibit superior predictability for patients in the PSA gray area, with the LogisticRegression model yielding the best prediction. The aforementioned predictive models can be used for actual clinical decision-making.​

## Introduction

​Prostate cancer (PCa) is an extremely common malignancy in men, its incidence being second only to lung cancer among all malignant tumors in men (1). Approximately 1.31 million new PCa cases are diagnosed each year globally, and nearly 10 million men currently have PCa. Out of these 10 million patients, approximately 700,000 suffer from metastatic PCa, which causes nearly 400,000 deaths each year, a death toll that is expected to more than double by 2040 (2). Therefore, it is becoming increasingly important to screen more accurately for PCa at an early stage.

Prostate specific-antigen (PSA) is a specific secretion of the prostate gland, which is a key marker of the initial diagnosis of the disease (3). However, this serum PSA level is influenced by various factors, such as ejaculation, acute prostatitis, urinary retention, catheterization, digital rectal examination (DRE), cystoscopy, and additional operations, thus affecting the accuracy of PSA diagnosis for patients (4). Clinically, PSA within the range of 4–10 ng/mL is defined as being in the gray zone (5). The possibility of PCa in the Chinese population with PSA in this range is about 25%, and the same is approximately 40% with the world population (6), and these patients are more likely to be missed for PCa or to have a non-essential puncture biopsy. In a study with 2426 patients, Xu B et al. found that the positive rate of PCa biopsy was only 25.77% for patients in the PSA gray zone (7). The diagnosis of PCa in the gray zone of PSA has become a key issue in urological research.

Machine learning can independently replicates human cognition and make decisions based on its perceived environment to achieve predetermined goals (8). Zhang S et al. proposed an interpretable deep learning framework for describing and interpreting human brain states (9). Furthermore, Lee C et al. developed a novel machine learning model for predicting non-metastatic PCa in men based on the Surveillance, Epidemiology, and End Results (SEER) database (10). ​Madhur Nayan et al. used machine learning to predict the progress of active PCa surveillance (11). Nevertheless, to date, only a few studies have used machine learning to forecast PCa with PSA in the gray zone. In the current study, four machine learning algorithms were compared.

## Materials and methods

### Data collection, the introduction of relevant variables, and handling of missing values

**​**Information was collected from December 01, 2014 to December 01, 2022 from the Department of Urology, The First Affiliated Hospital of Nanchang University, from patients with a pathological diagnosis of prostate hyperplasia or PCa (any PCa) and prior PSA of 4–10 ng/mL. According to the exclusion and inclusion criteria followed in this study, 756 patients were finally selected (596 patients with prostate hyperplasia and 160 patients with PCa). Age, free prostate-specific antigen (fPSA), total prostate-specific antigen (tPSA), fPSA/tPSA, prostate volume (PV), prostate-specific antigen density (PSAD), (fPSA/tPSA)/PSAD, absolute neutrophil count (NEUT), lymphocyte absolute count (LYM), platelet count (PLT), neutrophil absolute count/lymphocyte absolute count (NLR), absolute platelet count/absolute lymphocyte count (PLR), alkaline phosphatase (ALP), and prostate MRI results were recorded for the samples.

The MRI findings of the prostate were abnormal, the abnormality being defined as PI-RADS score >= 3. The PI-RADS score is the combined clinical presentation of mpMRI (multiparametric MRI) with DWI (diffusion-weighted imaging), T2WI (T2-weighted imaging of the prostate), and DCE (dynamic contrast enhancement). This score has considerable clinical significance in the diagnosis of PCa (12).

The cases were divided Into prostate hyperplasia and PCa groups based on the pathological findings. Most of the sample data of this study were relatively complete, and the primary limitation was the presence of PSA only in some samples that did not contain fPSA data. However, the missing data were padded using the random forest algorithm.

### Inclusion and exclusion criteria

​Inclusion criteria: 1, PSA in the range of 4–10 ng/mL; 2, a clear pathological diagnosis and no history of prostate surgery; 3, the relevant prediction metrics are complete. Exclusion criteria: 1, patients with acute prostatitis, acute urinary retention, within 48 h of cystoscopy and catheterization and within 1 week of DRE; 2, patients taking drugs that may affect outcomes, such as 5-alpha reductase inhibitors; 3, patients having more than two prostate punctures.

### Predictive variable screening

First, the overall distribution of the 14 variables included in this study was analyzed, and then a univariate logistic analysis was performed on each variable to screen for statistically significant predictors. After the Collinearity Diagnostics of predictor variables, multivariate logistic analysis was performed after screening out variables with no covariance and multiple indicators with independent influences. Machine learning models were selected to be incorporated for modeling in conjunction with actual clinical situations.​

### Building machine learning models

Sample imbalance in machine learning refers to an imbalance in the number of samples per category in a dataset. When building the model, it is biased towards samples with larger proportions, resulting in a low generalization ability. The ideal ratio of positive to negative samples for building the model is 1:1. In this study, the sample data for the PCa group: and prostate hyperplasia group was 160:596, and therefore, the sample showed an imbalance. Sample imbalance commonly originates from an over-sampling of under-represented categories or under-sampling of over-represented categories. Herein, over-sampling the PCa group, which is a small percentage of the total population, resulted in less accurate true data. To address this issue, the prostate hyperplasia group was randomly under-sampled, thereby altering the PCa group: prostate hyperplasia group to 160:320 (1:2).

**Figure 1:**
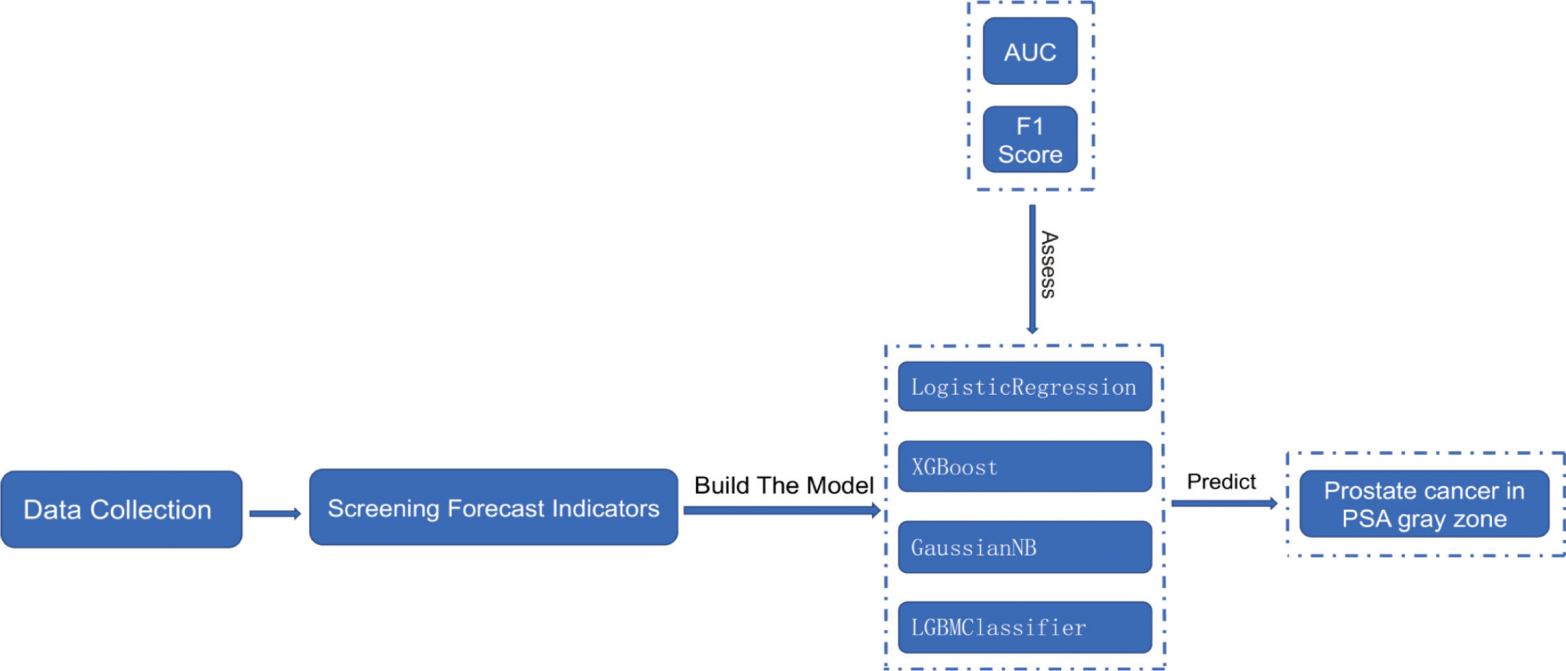
Flow chart of the cross-validation study.

To reduce model overfitting and ensure its stability and generalization ability, the total number of samples is considered to be low. The model is thus constructed using five-fold cross-validation on the dataset, which is divided into five parts. Each part is uncrossed and all the parts are of the same size. In descending order, four of the five replicas are selected as the training set and one as the validation set, and five separate model training and validation runs were performed. The final results of the five validation runs were averaged as the validation error of the model.​ The flow chart of this research is shown in **Figure 1**.

### Statistical analysis

The statistical analysis of the sample data was initially performed using SPSS 26.0 software. Continuous and categorical variables were expressed as mean ± standard deviation and percentage (%), respectively. Independent sample t-tests were performed to determine the differences between the groups. Differences in the AUC values of machine learning algorithms between groups were compared using De Lon’s non-parametric approach (4). The machine learning algorithms were constructed and compared using python version 3.10.​

## Results

After filtering according to the inclusion and exclusion criteria, a total of 765 cases were collected, including 596 in the prostate hyperplasia group and 160 in the PCa group. The case information for both groups is shown in **Table 1**.​ The comparison yielded statistically significant differences in the 11 predictors of tPSA, fPSA, fPSA/tPSA, PV, PSAD, (fPSA/tPSA)/PSAD, ALP, NLR, PLR, NEUT, and prostate MRI abnormalities. Age, LYM, and PLT were not statistically significant between the two groups. The overall mean of tPSA, PSAD, NLR, PLR, and ALP in the PCa group was greater than that in the prostate hyperplasia group (p < 0.05). Conversely, the overall mean of the fPSA, fPSA/tPSA, PV, and (fPSA/tPSA)/PSAD in the PCa group were smaller than those in the prostate hyperplasia group (p < 0.05).

**Table 1:** Distribution of variables and comparison of differences between the two groups of cases.

Projects	Prostatic hyperplasia group (N = 596)	Prostate cancer group (N = 160)	p-value
Age (years)	74.324 ± 6.984	74.250 ± 3.162	0.846
tPSA(ng/ml)	6.569 ± 1.502	7.061 ± 1.186	**<0.001**
fPSA(ng/ml)	1.501 ± 0.778	1.071 ± 0.338	**<0.001**
fPSA/tPSA	0.226 ± 0.095	0.154 ± 0.049	**<0.001**
PV(cm^3^)	60.089 ± 14.446	46.774 ± 12.594	**<0.001**
PSAD(ng/ml. cm^3^)	0.116 ± 0.038	0.166 ± 0.062	**<0.001**
(fPSA/tPSA)/PSAD	2.162 ± 1.057	1.085 ± 0.554	**<0.001**
NEUT(*10^9^/L)	4.730 ± 1.839	5.135 ± 0.713	**<0.001**
LYM(*10^9^/L)	1.396 ± 0.543	1.333 ± 0.612	0.235
PLT(*10^9^/L)	209.865 ± 65.421	212.410 ± 46.315	0.576
ALP(U/L)	89.432 ± 24.840	96.804 ± 19.687	**<0.001**
NLR	3.817 ± 2.088	4.511 ± 1.627	**<0.001**
PLR	166.984 ± 71.286	189.305 ± 84.711	**0.003**
MRI abnormalities[n(%)]			
no(0)	497(83.389%)	25(15.625%)	**<0.001**
yes(1)	99(16.611%)	135(84.375%)	

NEUT, absolute neutrophil count; LYM, lymphocyte absolute count; PLT, platelet count; ALP, alkaline phosphatase; NLR, neutrophil absolute count/lymphocyte absolute count; PLR, absolute platelet count/absolute lymphocyte count.

The bold numbers indicate the statistical differences.

**Figure 2:**
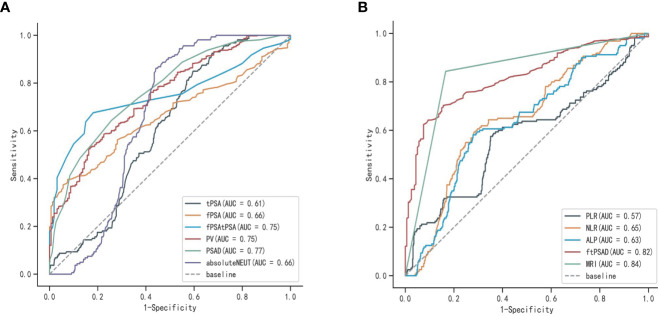
ROC curves for each predictor variable: **(A)** ROC curves of tPSA, fPSA, fPSA/tPSA, PV, PSAD, and ENEUT; **(B)** ROC curves of (fPSA/tPSA)/PSAD, ALP, NLP, PLR, and prostate MRI abnormalities.

Univariate logistic analysis was performed on the aforementioned statistically significant predictors, the results of which are presented in **Table 2**. In addition, the ROC curves for each predictor variable are shown in **Figure 2**. The AUC for the predictive efficacy of prostate MRI abnormalities was 0.839, and the AUCs for (fPSA/tPSA)/PSAD and PSAD were 0.818 and 0.769, respectively.

**Table 2:** Results of univariate logistic analysis.

Projects	AUC	Sensitivity	Specificity	Youden’s index	Optimal threshold
tPSA	0.609	0.850	0.404	0.254	6.010
fPSA5	0.664	0.396	0.925	0.321	1.570
fPSA/tPSA	0.748	0.676	0.819	0.495	0.190
PV	0.748	0.532	0.831	0.363	59.190
PSAD	0.769	0.644	0.743	0.387	0.140
NEUT	0.662	0.863	0.559	0.421	4.530
(fPSA/tPSA)/PSAD	0.818	0.624	0.925	0.549	1.700
ALP	0.632	0.581	0.721	0.303	95.650
NLR	0.650	0.594	0.716	0.310	4.160
PLR	0.574	0.588	0.638	0.225	170.520
MRI	0.839	0.844	0.834	0.678	1.000

NEUT, absolute neutrophil count; ALP, alkaline phosphatase; NLR, neutrophil absolute count/lymphocyte absolute count; PLR, absolute platelet count/absolute lymphocyte count.

​The results of the Collinearity Diagnostics for the above variables are given in **Table 3**. Among these, PSAD, NLR, PLR, NEUT, MRI, and ALP are variables with covariance index VIF values less than 10, indicating no covariance.​

**Table 3:** Covariance VIF values for each predictor variable.

Projects	VIF
fPSAtPSA	57.783
fPSA	35.782
ftPSAD	19.838
PV	11.935
tPSA	10.932
**PSAD**	**9.452**
**NLR**	**3.454**
**PLR**	**2.265**
**NEUT**	**2.039**
**MRI**	**1.113**
**ALP**	**1.059**

NEUT, absolute neutrophil count; ALP, alkaline phosphatase; NLR, neutrophil absolute count/lymphocyte absolute count; PLR, absolute platelet count/absolute lymphocyte count.

Bold numbers indicate items included in subsequent studies.

After including variables with VIF<10 for multivariate logistic analysis, PASD, and MRI variables were found to have p-values less than 0.001, and PASD and MRI were determined to be the independent risk factors for outcome variables (**Table 4**).

**Table 4:** Results of multivariate logistic analysis of predictor variables.

Projects	Estimate	SE	Z	p
NEUT	0.103	0.107	0.962	0.336
ALP	0.009	0.005	1.829	0.067
PLR	0.002	0.002	0.976	0.329
NLR	-0.056	0.116	-0.482	0.63
PSAD	25.198	3.187	7.906	**<0.001**
MRI	3.542	0.294	12.047	**<0.001**

NEUT, absolute neutrophil count; ALP, alkaline phosphatase; NLR, neutrophil absolute count/lymphocyte absolute count; PLR, absolute platelet count/absolute lymphocyte count.

The bold numbers mean statistically different. Bold numbers indicate items included in subsequent studies.

### Results of machine learning models for LogisticRegression; XGBoost; GaussianNB; LGBMClassifier

**Figure 3:**
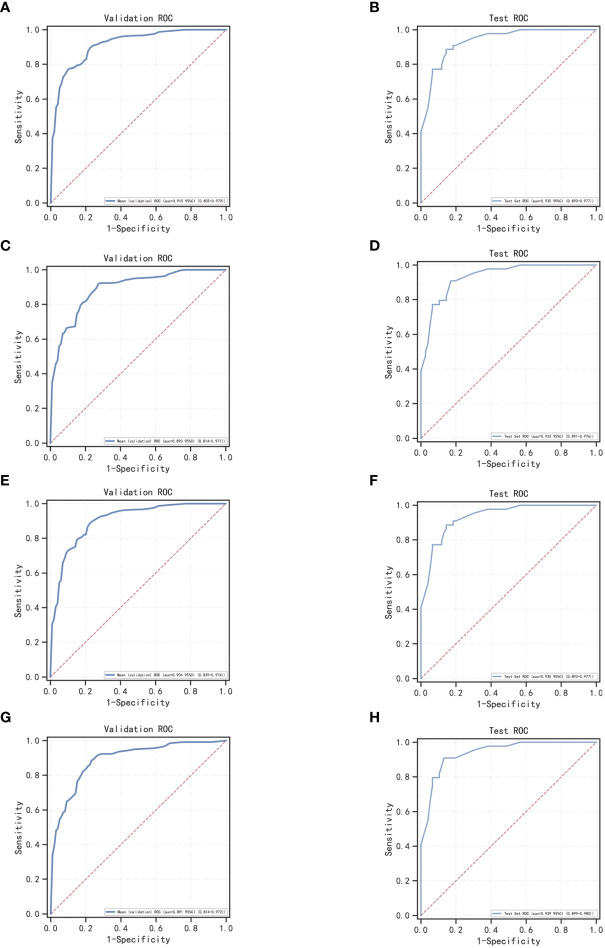
ROC curves for machine learning models for LogisticRegression, XGBoost, GaussianNB, and LGBMClassifier; **(A, C, E, G)** ROC curves of four machine learning methods on the validation set; **(B, D, F, H)** ROC curves of four machine learning methods applied on the test set.

In the overall sample, N = 120 cases (25.00%) were randomly selected as the test set. The remaining samples were used as the training set for five-fold cross-validation. ROC curves for the validation and test sets of LogisticRegression, XGBoost, GaussianNB, and LGBMClassifier are shown in **Figure 3**.​ The AUC values of their validation sets were 0.918 (0.855–0.979), 0.893 (0.814–0.971), 0.906 (0.839–0.974), and 0.893 (0.814–0.972), respectively. Furthermore, the AUC values of the measured sets were 0.935 (0.893–0.977), 0.933 (0.891–0.976), 0.935 (0.893–0.977), and 0.939 (0.899–0.980), respectively (**Table 5**). Since the AUC of the validation set did not exceed that of the test set, or the excess ratio was less than 10%, the fit was considered successful. All the aforementioned models can be used for this dataset.

**Table 5:** Results of the validation and test sets of four machine learning models.

	Data set	AUC(95% Cl)	Accuracy	Sensitivity	Specificity	Positive predictive value	Negative predictive value	F1 score
LogisticRegression	Validation set	0.918 (0.855–0.979)	0.811	0.88	0.844	0.666	0.914	0.758
	Test set	0.935 (0.893–0.977)	0.875	0.886	0.855	0.872	0.877	0.879
XGBoost	Validation set	0.893 (0.814–0.971)	0.792	0.914	0.762	0.659	0.88	0.765
	Test set	0.933 (0.891–0.976)	0.85	0.909	0.829	0.81	0.872	0.856
GaussianNB	Validation set	0.906 (0.839–0.974)	0.794	0.906	0.799	0.64	0.922	0.749
	Test set	0.935 (0.893–0.977)	0.875	0.886	0.855	0.872	0.877	0.879
LGBMClassifier	Validation set	0.893 (0.814–0.972)	0.8	0.914	0.766	0.67	0.893	0.771
	Test set	0.939 (0.899–0.980)	0.883	0.909	0.868	0.875	0.888	0.892

### Categorical multi-model construction and the comparison of respective ROC curves

**Figure 4:**
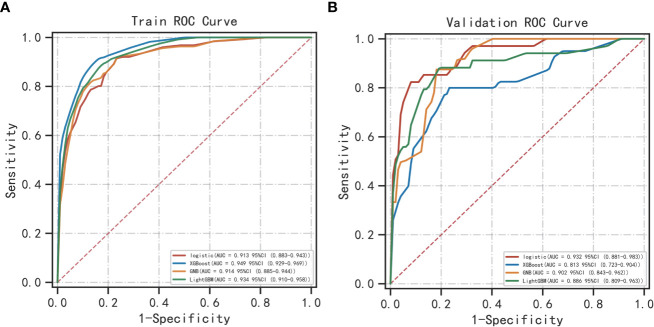
ROC curves of categorical multivariate models: **(A)** Training sets; **(B)** Validation sets.

**Figure 5:**
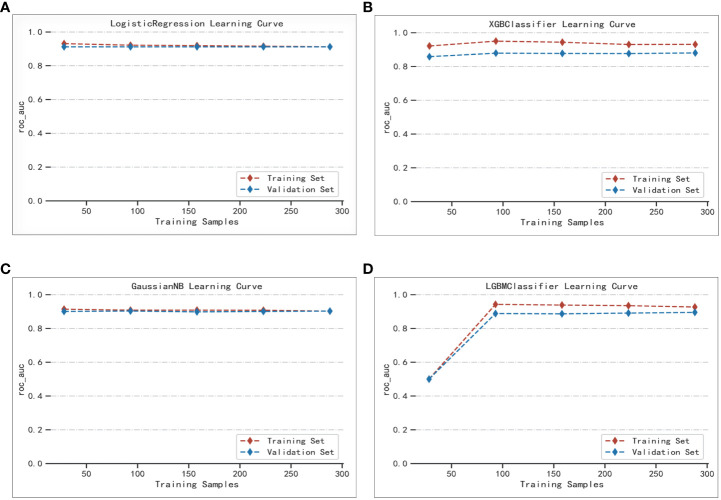
Extent of matching of the validation and test sets of the four machine learning methods: **(A)** LogisticRegression; **(B)** XGBoost; **(C)** GaussianNB; **(D)** LGBMClassifier.

The best performer in the training set was XGBoost, while that in the validation set was LogisticRegression (both ranked according to AUC) (**Figure 4**). A comparison of the training and validation sets shows that the results do not match (**Table 6**). XGBoost most likely resulted in overfitting, while LogisticRegression demonstrated relatively excellent stability and is therefore recommended as the optimal prediction model. From the change of AUC during the training of each model, it is observed that the AUCs of the training and validation sets are finally stable and well-matched above 0.8 (**Figure 5**).

**Table 6:** ​Results of the machine learning categorical multi-model.

Training sets							
Models	AUC(95% CI)	Accuracy	Sensitivity	Specificity	Positive predictive value	Negative predictive value	F1 score
LogisticRegression	0.913 (0.883–0.943)	0.815	0.913	0.771	0.663	0.935	0.768
XGBoost	0.949 (0.929–0.969)	0.872	0.900	0.852	0.752	0.942	0.819
GaussianNB	0.914 (0.885–0.944)	0.854	0.824	0.863	0.790	0.890	0.806
LGBMClassifier	0.934 (0.910–0.958)	0.852	0.881	0.833	0.732	0.928	0.799
Validation sets							
Models	AUC(95% CI)	Accuracy	Sensitivity	Specificity	Positive predictive value	Negative predictive value	F1 score
LogisticRegression	0.932 (0.881–0.983)	0.792	0.824	0.919	0.652	0.920	0.728
XGBoost	0.813 (0.723–0.904)	0.771	0.800	0.768	0.737	0.793	0.767
GaussianNB	0.902 (0.843–0.962)	0.813	0.875	0.819	0.600	0.909	0.712
LGBMClassifier	0.886 (0.809–0.963)	0.833	0.882	0.806	0.725	0.911	0.796

### DeLong test for categorical multi-model AUC values

The DeLong test is used to verify whether the AUC values of the two sets of machine learning models are statistically different (**Table 7**). From **Table 7**, it is evident that the difference between LogisticRegression and the three machine learning models XGBoost, GaussianNB, and LGBMClassifier is significant (p<0.001). The remaining machine learning models do not differ from each other in a statistically significant way.

**Table 7:** ​Results of Delong test.

LogisticRegression	XGBoost	GaussianNB	LGBMClassifier
NaN	<0.001	<0.001	<0.001
**<0.001**	NaN	0.667	0.769
**<0.001**	0.667	NaN	0.429
**<0.001**	0.769	0.429	NaN

NaN, Not a Number.

The bold numbers indicate statistically different.

## Discussion

PCa is one of the most prevalent cancers in the world (13). Approximately 25% of patients with PSA between 4.0 and 10.0 ng/mL were found to have PCa, compared with approximately 40 percent in the United States​ (14). Currently, artificial intelligence is widely used in medicine. Machine learning algorithms accept predictor variable metrics as inputs and provide variable metrics as outcomes and build appropriate machine learning models. Machine learning can also predict the current input data based on an existing model, which considers the prediction metrics and considerably improves the prediction accuracy. This technique exhibits a better prediction performance than individual prediction metrics, so its application prospects are numerous (15, 16). ​Related studies have demonstrated the tremendous value of machine learning models for PCa diagnosis and the assessment of prognosis (17–20). For example, Satoshi Nitta et al. used age, PSA, PV, and urine leukocyte count as predictors to build a PCa prediction model based on Random Forest, Support Vector Machines (SVM), and artificial neural networks (ANNs) (21). Wouter Bulten et al. built a machine learning model to grade the Gleason score and compared it with professional pathologists. The results indicated that this model had diagnostic values comparable to professional pathologists (22). ​Simon P Hood et al. developed an ensemble machine learning predictive model to predict low and high-risk PCa (23). Further, Patrick Schelb et al. classified PCa based on MRI by machine learning combined with PI-RADS scoring (24). ​Similarly, numerous studies of machine learning in the classification of PCa and prostate enlargement have been conducted to date (25–30). Fewer studies, however, have targeted patients in the gray zone of PSA. In addition, the machine learning model built for the overall range of PSA is not well suited for PCa with PSA in the gray zone. Meanwhile, in their study of the utility and limitations of cadaveric examination in organ transplantation, Desley Neil et al. (31, 32) found that although PCa is incidentally detected at the cadaveric examination of organ donors, the likelihood of this disease increases with donor age. Thus, this poses new requirements and challenges for PCA diagnosis. It is to be noted that machine learning is particularly important in the diagnosis of PCa in organ transplantation.

The differences in the 11 predictors of tPSA, fPSA, fPSA/tPSA, PV, PSAD, (fPSA/tPSA)/PSAD, ALP, NLR, PLR, NEUT#, and prostate MRI abnormalities were statistically significant as seen in the general patient data. Notably, while the mean age of the PCa group was greater than that of the prostate enlargement group, the difference was not statistically significant. The mean PV in the PCa group was also smaller than that in the prostate hyperplasia group, which is consistent with the overall range of the relevant literature in which PV in PCa is larger than that in prostate hyperplasia. This may be owing to the error caused by the limited sample size. In the univariate logistic analysis of predictive variables, the AUC of prostate MRI abnormalities to determine PCa was 0.84. This predictor has an elevated predictive efficacy for PCa. In addition, the prediction performances for (fPSA/tPSA)/PSAD and PSAD are also better, and this is consistent with the findings of ZhangYiyan et al. (30). Since PSA is influenced by numerous factors and the variability is not particularly significant when PSA is in the gray zone, the use of PSAD is more valuable than that of PSA in terms of predictive performance. As claimed by S. M. Bruno et al. (33) in a prospective study, PASD is a strong predictor of PCa because it can exclude the influence of elevated PSA due to prostatitis on the diagnosis. Therefore, the inclusion of PASD in this study is also essential. Since all fPSA/tPSA and (fPSA/tPSA)/PSAD are derived indicators, they exhibited covariance in the covariance analysis. After multivariate logistic analysis of tPSA, NLR, PLR, ALP, PSAD, and prostate MRI predictors, the difference in the predictive index of PSAD and prostate MRI abnormalities was significant (p<0.001), and PSAD and prostate MRI abnormalities were the independent risk factors for PCa with PSA in the gray zone. Owing to the development of reflectomics, the future application of radiomics in the PCa diagnosis bears potential, as described by M. Ferro et al. (34) in their review on the application of radiomics in PCa. Although the study of radiomics is primarily focused on mpMRI, it integrates several other factors to render the PCa diagnosis more accurate and sensitive. Unfortunately, only mpMRI was used as a predictor in the current study.

Herein, the validation sets for the four machine learning models have AUC values of 0.918, 0.893, 0.906, and 0.893, accuracies of 0.811, 0.792, 0.794, and 0.800, and F1 scores of 0.758, 0.765, 0.749, and 0.771, respectively. This demonstrates the better performance of LogisticRegression over that of GaussianNB and LGBMClassifier on the validation sets, and the performance of LGBMClassifier is comparable to that of XGBoost. In the classification multi-model validation, the LogisticRegression model demonstrated the best predictive efficacy (AUC = 0.932) and the difference with XGBoost, GaussianNB, and LGBMClassifier was statistically significant (p<0.001). ​Bermejo et al. showed that a single indicator, PSA, is not adequately effective in diagnosing PCa. Nevertheless, when logistic regression and decision tree models are built by combining age, DRE, PSA, mpMRI, and other relevant indicators, both models achieve better accuracy (35). All the aforementioned studies, although different from the subject of this study, illustrate the remarkable value of machine learning models in PCa diagnosis and prediction capabilities.​ Previous studies on the diagnosis of PCa were compared using other predictive methods, such as I. M. Perez et al.’s biparametric MRI (bpMRI) as the main predictor (36). The AUC values of the resulting prediction models in the development and validation queues were 0.83 (0.77–0.89) and 0.80 (0.75–0.85), lower than those of the machine learning models in this study. In another study, S. Parekh et al. (37) used three predictive model coves (PROMOD, ERSPC RC, and ANN) with AUC values of any PCa (0.82 vs 0.70 vs 0.90) and csPCa (0.82 vs 0.78 vs 0.92). All these values are lower than the AUC values of the machine learning models in the current study, especially the LogisticRegression algorithm.

Although a total of 756 cases were included in this study, the sample size is still limited and prone to bias. Moreover, although the sample number ratio of the prostate hyperplasia group to the PCa group was maintained at 1:2 to ensure a balanced sample, the total sample size being modest, it was prone to problems such as overfitting and poor generalization ability. Therefore, an increase in sample size was needed to ensure the reliability of the results. In addition, information about prostate puncture, such as the number of needles in the puncture, transperineal, or transrectal, and whether MRI ultrasound fusion was performed or not (38), are not recorded in this study, which should be included in subsequent studies.

## Conclusion

​ Machine learning prediction models based on LogisticRegression, XGBoost, GaussianNB, and LGBMClassifier algorithms for PCa with PSA in the gray region exhibit remarkable predictive power, and the LogisticRegression machine learning model has higher predictive performance than the rest of the models. The aforementioned machine learning models can be used for practical clinical decision-making to assist physicians in the personalized diagnosis and treatment of patients.​

## Data availability statement

The original contributions presented in the study are included in the article/supplementary material. Further inquiries can be directed to the corresponding authors.

## Ethics statement

Ethical review and approval were not required for the study on human participants in accordance with the local legislation and institutional requirements. Written informed consent for participation was not required for this study in accordance with the national legislation and institutional requirements.

## Author contributions

The data was collected by XZ. TL conceived and wrote the manuscript. XZ and TL worked together on the tables and figures. RC provides guidance in statistical analysis. RC and BF provided input into the revision of the manuscript. XD made changes to the language of the manuscript. All authors contributed to the article and approved the submitted version.
